# The Prevalence of Virulent and Multidrug-Resistant Enterococci in River Water and in Treated and Untreated Municipal and Hospital Wastewater

**DOI:** 10.3390/ijerph18020563

**Published:** 2021-01-11

**Authors:** Anna Gotkowska-Płachta

**Affiliations:** Department of Water Protection Engineering and Environmental Microbiology, The Faculty of Geoengineering, University of Warmia and Mazury in Olsztyn, Prawocheńskiego 1, 10-719 Olsztyn, Poland; aniagp@uwm.edu.pl

**Keywords:** enterococci, multidrug-resistant (MDR), virulence, vancomycin, river water, wastewater

## Abstract

The aim of this study is to describe the drug resistance and virulence of enterococci in river water sampled downstream (DRW) and upstream (URW) from the wastewater discharge point, to determine the pool of virulent and drug-resistant enterococci in untreated wastewater (UWW) and the extent to which these bacteria are eliminated from hospital wastewater (HWW) and municipal wastewater treated (TWW) by biological and mechanical methods in a wastewater treatment plant (WWTP). A total of 283 strains were identified with the use of culture-dependent methods and PCR, including seven different species including *E. faecalis* and *E. faecium* which were predominant in all analyzed samples. Majority of the strains were classified as multidrug resistant (MDR), mostly on streptomycin and trimethoprim. Strains isolated from wastewater and DRW harbored *van* genes conditioning phenotypic resistance to vancomycin, the highest percentage of vancomycin-resistant strains (57.0%), mostly strains harboring *van*C1 genes (27.6%), was noted in TWW. More than 65.0% of the isolated strains had different virulence genes, the highest number of isolates were positive for cell wall adhesin *efa*A and sex pheromones *cob*, *cpd,* and *ccf* which participate in the induction of virulence. Many of the strains isolated from TWW were resistant to a higher number of drugs and were more virulent than those isolated from UWW and HWW. The enterococci isolated from DRW and wastewater were characterized by similar multidrug resistance and virulence profiles, and significant correlations were observed between these groups of isolates. These findings suggest that pathogenic enterococci are released with TWW and can spread in the river, pose a serious epidemiological threat and a risk to public health.

## 1. Introduction

Wastewater is treated to reduce pollutant concentrations to environmentally safe levels. However, various compounds and pathogenic fecal bacteria, including antibiotic-resistant bacteria (ARB) and antibiotic-resistance genes (ARGs), are evacuated to surface water bodies with treated wastewater [1,2,3]. These micropollutants influence the trophic status, microbiological quality, and epidemiological safety of natural ecosystems. Bacteria and their metabolites are evacuated with wastewater, and they disrupt the natural balance of microbiota which colonize aquatic systems, actively participate in the biogeochemical cycle, restore and maintain ecological balance in water ecosystems. The inflow of allochthonous microorganisms as well as other organic and inorganic pollutants to aquatic ecosystems disrupts the ecological balance and exerts a negative impact on a given biotope [4,5,6]. Gram positive enterococci rapidly evolve and spread in the environment. These oval-shaped bacteria often occur in pairs or short chains, and they are able to grow in a wide range of temperatures (10 °C to 45 °C), pH of 9.6, and NaCl concentration of up to 6.5% [7,8]. Enterococcus strains can be both pathogenic and commensal. Commensal bacteria colonize the digestive tract of humans and animals, and they are not highly virulent. Enterococci are fecal indicator bacteria that are used to assess fecal pollution of the natural environment. Enterococci are also indirect indicators of the risks associated with the spread of other pathogens in aquatic ecosystems and the transmission of waterborne diseases [9,10,11,12]. Therefore, these bacteria are used to monitor water quality and detect sanitary and epidemiological threats worldwide. In non-contaminated waters, enterococcus counts generally do not exceed several to less than 20 colony-forming units in 100 mL of water. However, the inflow of treated wastewater, surface runoffs from agricultural land, and pollutants from recreational areas considerably increases enterococcus counts in aquatic systems [4]. Enterococcus counts ranged from 4.5 × 10^1^ to 1.2 × 10^4^ cfu 100 mL^–1^ in the rivers of the Seine watershed [13]. The abundance of enterococci in less polluted rivers was similar to that in nonpoint source pollution loads in forests, and their concentrations in the most heavily contaminated rivers were similar to those in treated wastewater. In untreated municipal wastewater, the abundance of fecal enterococci and bacteria of the family *Enterobacteriaceae* was determined in the range of 4 to 8 log cfu mL^−1^ [6,13,14]. Wastewater treatment reduces the counts of indicator microorganisms by even several orders of magnitude, but large amounts of these bacteria reach water receptacles with the evacuated effluents and compromise the microbiological safety of surface water bodies.

Selected species of the family Enterobacteriaceae are used in the production of probiotics and starter cultures in the food-processing industry and veterinary medicine [15,16]. However, because of the growing levels of drug resistance, enterococci are increasingly often classified as alert pathogens in the hospital environment. These strains are particularly dangerous for patients with respiratory diseases and patients receiving broad-spectrum antimicrobial drugs [17,18,19]. *Enterococcus faecalis* and *E. faecium* pose the greatest epidemiological risk, whereas *E. avium*, *E. casseliflavus*, *E. durans*, *E. gallinarum*, and *E. raffinosus* are less likely to cause infections in hospitals [20,21]. Recent years have witnessed a steady increase in the abundance of vancomycin-resistant *E. faecium* belonging to the ESKAPE group of bacteria (*Enterococcus faecium*, *Staphylococcus aureus*, *Klebsiella pneumoniae*, *Acinetobacter baumannii*, *Pseudomonas aeruginosa*, *Enterobacter* spp.) responsible for hospital infections [18,22]. Enterococci cause various diseases, including urinary tract infections, infectious diseases in infants, postoperative infections, infections of the central nervous system, and endocarditis [23,24,25]. Virulent and vancomycin-resistant enterococci (VRE) are particularly important from the medical point of view. Enterococcus strains identified both in and outside of hospitals are increasingly often resistant to vancomycin. Until recently, vancomycin was regarded as the most potent antimicrobial drug, but it is no longer effective against the VRE strains [18,26]. Several resistance phenotypes have been identified in enterococci depending on their resistance to vancomycin and teicoplanin (*van*A B, C, D, E, G, L, M, and *van* N). The most prevalent phenotypes, *van*A and *van*B, have been described mainly in *E. faecalis* and *E. faecium*, and phenotype *van*C1—in *E. casseliflavus* and *E. gallinarum* [27,28,29]. In 2018, the highest number of clinical VRE isolates of *E. faecium* were determined in Romania (40.3%), Ireland (40.2%), and Poland (35.8%), (ECDC 2020). Vancomycin-resistant enterococci are isolated in various countries (Ethiopia—4.8%, North America—21%, Asia—24%, Europe—20%), and their prevalence is influenced by the applied isolation method, time of analysis, and the size of the studied population [30,31]. Some species harbor virulence factors, including cytolysin which facilitates cell lysis (*cyl*A), aggregation substance (AS), gelatinase which hydrolyzes gelatin, casein, and hemoglobin (*gel* E), hyaluronidase which increases the invasiveness of bacteria (*hyl*), endocarditis antigen (*efa*A), factor-encoding surface protein that participates in biofilm formation (*esp*), and sex pheromones that carry ARGs (*cob*, c*pd*, and *ccf*) [32,33,34]. According to the Polish National Program for the Protection of Antibiotics (NPOA) and the European Center for Disease Prevention and Control (ECDC), the prevalence of antibiotic-resistant *E. faecalis* and *E. faecium* strains continues to increase around the world. In recent years, the spread of virulent and multidrug-resistant (MDR) enterococci was also observed outside healthcare facilities in various environments [13,18,35,36]. The scale and sources of bacterial dissemination can differ, and they have not been fully elucidated. Human activities, in particular the overuse of antibiotics in veterinary medicine, livestock production (mostly avoparcin which is used as a feed additive), agriculture, and human medicine have contributed to the expansion of pathogenic strains [37]. The plastic genome enables enterococci to exchange genes on mobile genetic elements such as transposons and plasmids. These microorganisms can accumulate and transfer ARGs to other bacteria. As a result, contact with infected livestock, handling of food products of animal origin, and runoffs from agricultural areas fertilized with manure or slurry can promote the transfer of MDR strains, including VRE [38,39,40]. Hospitals are also hotspots of ARB and ARGs because of a large number of antibiotic treatments and the presence of significant clinically resistant pathogenic bacteria. Municipal WWTPs that process municipal and hospital wastewater can also act as critical points in the transfer of resistant strains to the environment, and transfer of antibiotic resistance. Multidrug-resistant strains, including VRE, are increasingly often identified in wastewater and wastewater receptacles [14,36,37,41]. Research into the prevalence of ARB and ARGs in rivers and streams plays a particularly important role. Microbiological pollutants are carried along the river continuum, and resistance genes can be transmitted to native bacteria colonizing the aquatic environment. As a result, rivers become potential reservoirs of ARB and ARGs, and they pose a significant sanitary and epidemiological threat for users of water.

In the present study, samples of river water were collected 500 m downstream and upstream from the wastewater discharge point to determine the sources of contamination with virulent and MDR enterococci. The prevalence of bacteria was also determined in hospital wastewater and in untreated and treated wastewater. The extent to which virulent and MDR enterococci were eliminated by biological and mechanical treatment methods in the WWTP was analyzed. Sampling sites were compared against markers of bacterial virulence and multidrug resistance. The main sources of contamination with enterococci should be identified in lotic ecosystems to support their protection and to prevent the spread of virulent and MDR bacteria in the environment.

In the light of Polish regulations, treated wastewater does not have to be disinfected before it is evacuated to surface water bodies, which promotes the transfer of pathogenic enterococci and their antibiotic resistance genes (ARGs) to the environment and poses health risks for humans and animals. In this context, the results of this study contribute important knowledge and can be used to plan effective measures to minimize bacterial emissions to the environment and reduce epidemiological risks.

## 2. Materials and Methods

### 2.1. Sampling Sites and Methods

The study was conducted in the Łyna Municipal Wastewater Treatment Plant in Olsztyn, the capital of the Region of Warmia and Mazury in north-eastern Poland. The plant is equipped with biological and mechanical wastewater treatment systems and has a daily processing capacity of around 32,000 m^3^. Effluents from three hospitals in Olsztyn account for around 2% of the treated wastewater. Technological scheme of the wastewater treatment plant is presented in Figure S1 (Supplementary Materials). These hospitals do not operate on their own WWTPs or wastewater disinfection systems. Each year, they admit around 46,000 patients on average. Wastewater is produced by hospital laboratories, departments, and wards, including surgery, oncology, cardiology, urology, gynecology, ophthalmology, anesthesiology, dermatology and otolaryngology wards, and intensive care units [42].

In the studied WWTP, wastewater is treated in mechanical and biological systems with the involvement of the activated sludge technology and chemical processes. Treated wastewater is not disinfected through chlorination or exposure to UV light before it is discharged to the river. Mechanical treatment involves preliminary purification (screening, sedimentation of mineral suspensions in grit chambers, and organic suspensions in preliminary sedimentation tanks). Pretreated wastewater is pumped to the biological treatment station where nitrogen, phosphorus, and carbon are removed in a series of nitrification, denitrification, and dephosphatation processes. The sediment is separated in sedimentation tanks, and treated wastewater is evacuated directly to the Łyna River which is one of the main lowland watercourses in the southern watershed of the Baltic Sea (northern Poland). The Łyna River has been characterized in detail by Gotkowska-Płachta et al. [6].

Samples of river water were collected 500 m downstream (DRW) and upstream (URW) from the wastewater discharge point. Hospital wastewater (HWW) was sampled at the place of generation, directly from hospital sewer sumps (before reaching the main sewer pipe). Samples of untreated wastewater (UWW) were collected directly behind the grating screen, and treated wastewater (TWW) was sampled from the outflow pipe before it was evacuated to the Łyna River. River water and wastewater samples were collected at approximately eight-week intervals over a period of 12 months, taking into account the research seasons. Municipal wastewater was sampled 22 h after the collection of HWW samples to account for the time needed to reach the WWTP. Water and wastewater samples were collected into sterile glass containers (1000 mL). River water was sampled at a depth of 0.3–0.5 m. A total of 16 river water samples and 40 wastewater samples (8 UWW samples, 8 TWW samples, and 24 HWW samples) were collected in the analyzed period. The samples were transported to the laboratory in insulated containers at a temperature of 4 °C. They were analyzed in the laboratory within 24 h after transport.

### 2.2. Determination of Physicochemical Parameters

The following physicochemical parameters of river water and wastewater samples were determined: temperature (°C) and pH. The measurements were conducted with the YSI 556 Multiprobe System (MPS) with an accuracy of ±0.1 °C and ±0.01 pH.

### 2.3. Isolation of Enterococci from Samples of River Water and Wastewater

Enterococci were enumerated by membrane filtration on Slanetz and Bartley (SB) medium after 48 h of incubation at a temperature of 36 ± 2 °C. The results were validated on Bile Aesculin Azide Agar (Marck) after 2 h of incubation at a temperature of 44 ± 0.5 °C according to Polish Standard [43]. The emerged colonies were counted and expressed in colony-forming units (cfu) in 100 mL of water or wastewater. Ten representative colonies were selected from each sample, plated on brain–heart infusion (BHI) broth and incubated at 37 °C for 24–48 h. Pure strains were initially identified to the genus *Enterococcus* based on the results of Gram stains, the catalase test, bacterial proliferation in BHI broth with the addition of 6.5% NaCl, and esculin hydrolysis into esculentin on Bile Aesculin Azide Agar (Merck) [44]. A total of 430 tested strains were preliminarily classified as *Enterococcus* spp. The isolates were stored on LB Miller medium (Merck) with 10% glycerol at a temperature of −80 °C until further analysis.

### 2.4. Identification of Enterococci to Genus and Species Level

The isolated strains were identified to be the genus *Enterococcus* by identifying the *tuf* gene encoding elongation factor EF–Tu, which has a strongly conserved sequence in the analyzed bacteria. An internal primer targeting a fragment (*sod*A *int*) of the *sod*A gene which encodes manganese dismutase was designed. Bacterial species were identified by amplifying *ddl* genes that encode D–alanyl–D–alanine ligase (D–Ala–D–Ala). Primers specific for this fragment of the gene supported the identification of *Enterococcus faecalis* and *Enterococcus faecium* which play a key role in human and animal infections. Strains belonging to *E. durans, E. hirae, E. avium,* and *E. gallinarum/E. casseliflavus* were identified. Primer sequences (synthesized by Genomed), product size, and PCR conditions are presented in Table S1—Supplementary Materials. The reactions were controlled with the use of the following reference strains: *E. faecalis* ATCC 29212, *E. faecium* ATCC 19434, *E. casseliflavus* ATCC 49605; *E. gallinarum* ATCC700425, *E. durans* ATCC 6056, and *E. hirae* ATCC 8043.

### 2.5. Determination of Enterococcus Susceptibility to Antimicrobial Drugs

All enterococcus isolates were analyzed for susceptibility to 13 antimicrobial drugs by the disc diffusion method with the use of antibiotics produced by Oxoid (Baningstoke, Hampshire, England). The selected antimicrobials belonged to 10 groups of antibiotics that are widely used in medicine and agriculture: (1) penicillins: ampicillin (AMP—2 μg); (2) carbapenems: imipenem (IPM—10 μg), (3) aminoglycosides: gentamicin (GEN—30 μg); streptomycin (S—300 μg); (4) glycopeptides: teicoplanin (TEC—30 μg), vancomycin (VAN—5 μg); (5) streptogramins: quinupristin/dalfopristin (QD—15 μg); (6) glycylcyclines: tigecycline (TGC—15 μg); (7) oxazolidinones: linezolid (LZD—10 μg); (8) tetracyclines: doxycycline (DO—30 μg); (9) fluoroquinolones: ciprofloxacin (CIP 5—5 μg), (10) chemotherapeutics: nitrofurantoin (NIT—100 μg), trimethoprim (W5—5 μg). The analyzed strains were classified as multidrug resistant (MDR) and extensively drug resistant (XDR). Multidrug-resistant bacteria were defined as microorganisms that were resistant to at least one antibiotic from at least three groups of antimicrobial drugs targeting a given species. Extensively, drug-resistant bacteria are defined as microorganisms resistant to at least one antibiotic in all but two antibiotic groups targeting a given species [45]. The sensitivity of the tested strains was determined on Mueller–Hinton agar (bioMerieux). Bacteria were incubated at 37 °C for 16–18 h, and resistance to vancomycin was determined after 24 h of incubation. The diameter of the inhibition zone was expressed in millimeters, and strains were classified as susceptible or resistant. The reference strain *Enterococcus faecalis* ATCC 29212 was used as the control strain. Susceptibility analyses were based on the guidelines of the European Committee on Antimicrobial Susceptibility Testing (EUCAST 2012) [46] and Clinical and Laboratory Standards Institute (CLSI 2012) [47].

### 2.6. Isolation of Genomic DNA and PCR Conditions

To isolate genomic DNA, a single bacterial colony was collected from the agar plate (BHI agar) after 24 h of incubation, suspended in Tris–EDTA buffer, digested with lysozyme (0.6 mg L^−1^) and heated at 95 °C for 10 min (QBD2 block heater, Grant). The colony was centrifuged at 5000 rpm for 5 min at 4 °C, and the concentration and quality of the isolated DNA was determined using a spectrophotometer (Eppendorf BioSpectrometer^®^ kinetic, Eppendorf, Hamburg, Germany). If DNA was of poor quality, it was isolated again with the Genomic Mini AX Bacteria Mini Kit (SPIN) (A&A Biotechnology, Gdynia, Poland) according to the manufacturer’s instructions. Genomic DNA was isolated in triplicate and stored at a temperature of −20 °C until further analysis. Single and multiplex polymerase chain reactions (PCR) were carried out in a thermal cycler (Eppendorf, Mastercycler Family, Hamburg, Germany). The total volume of the reaction mix was 20–50 μL for different reactions and the evaluated bacterial properties. The phases of the amplification process (denaturation, hybridization, and elongation) were adapted to a given reaction type (Tables S1–S3, Supplementary Materials). Amplified PCR products were separated and visualized on 1.5% agarose gel stained with ethidium bromide (1 mg L^−1^) (Sub–Cell^®^ GT system, Bio–Rad, CA, USA). 100–1000 bp and 142–3794 bp DNA ladders (A&A Biotechnology, Gdynia, Poland) were used as molecular size markers. DNA bands were visualized under UV light with a gel documentation kit (DGelScan, Kucharczyk, Warsaw, Poland). Randomly selected amplicons were sequenced and identified in the BLAST program available on the website of the National Center for Biotechnology Information (www.ncbi.nlm.nih.gov/BLAST) to confirm the identity of the isolated Enterococcus strains.

### 2.7. Identification of Enterococci and Detection of Vancomycin Resistance Genes and Virulence Factors by PCR

The isolated strains were identified to species level, and vancomycin resistance genes and virulence factors were detected by single and multiplex PCR. The most prevalent vancomycin resistance genes—*van*A, *van*B, *van*C1, and *van*C2/C3—were identified. The ten most important enterococcus virulence factors were identified: *cyl*A—cytolysin toxin also known as hemolysin; *hyl*—hyaluronidase enzyme; *ace*—collagen–binding surface protein; *Efa*A—cell wall adhesins; *gel*E—gelatinase; *as—*aggregation substance; *esp*—extracellular surface protein; *cpd*, *cob*, *ccf*—sex pheromones that participate in the exchange of genetic material between strains. Primer sequences (synthesized by Genomed), product size, and PCR conditions are presented in Tables S1–S3—Supplementary Materials.

### 2.8. Statistical Analysis

Statistical analyses were performed in the Statistica 13.2 program software package (StatSoft Inc., 1984–2019, Tulsa, OK, USA) at a significance level of 0.05. Spearman’s rank correlation coefficient was calculated to determine the correlations between the analyzed physicochemical parameters, total enterococcus counts, and the number of MDR and virulent strains. Because of abnormally distributed data, the Kruskal–Wallis (KW) test, a non-parametric version of classical one-way analysis of variance ANOVA, was used to determine the differences in enterococcus counts in wastewater and river water depending on the time of the sample collection and the type of investigated wastewater. A heatmap was generated, and a cluster analysis was performed with the use of Ward’s hierarchical cluster method in RStudio (Version 1.2.5033) using “heatmap.2” and “gplots” packages.

## 3. Results

### 3.1. Physicochemical Parameters

The physicochemical parameters of river water and wastewater samples are presented in Table 1. The average pH of wastewater samples was similar during the entire period of the study in the range of 8.35 (HWW) to 8.40 (TWW). The average temperature of wastewater samples did not exceed 16 °C. The temperature in most URW sampling sites was above 20 °C in summer and below 10 °C in winter.

### 3.2. Microbial Counts in Wastewater and River Water Determined by the Culture-Dependent Method

**Figure 1:**
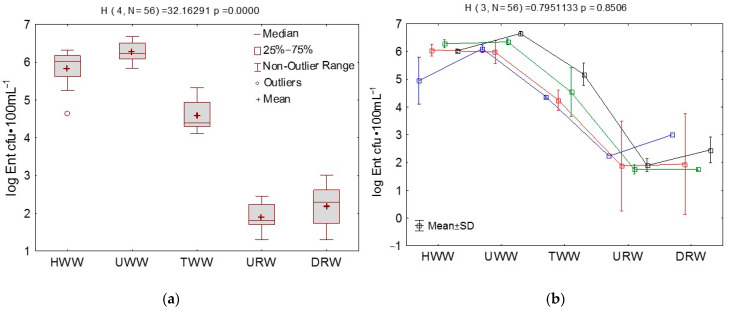
Enterococcus counts [log Ent cfu·100 mL^−1^] determined by the culture-dependent method in river water and wastewater sampled from different (**a**) sites and (**b**) seasons during the study. Kruskal–Wallis test (ANOVA), SD—standard deviation; *N*—number of samples; *p*—significance level.

Enterococcus counts ranged from 1.30 log cfu·100 mL^−1^ in URW and DRW to 6.68 log cfu·100 mL^−1^ in UWW. The highest average bacterial counts were noted in UWW and HWW (6.27 and 5.83 log cfu·100 mL^−1^, respectively). In URW and DRW samples, the average enterococcus counts did not exceed 2.2 log cfu·100 mL^−1^ and were several orders of magnitude lower than in wastewater (Figure 1). Bacterial counts were highest in HWW in summer (6.28 log cfu·100 mL^−1^) and in TWW and UWW in fall (5.18 and 6.65 log cfu·100 mL^−1^, respectively). In URW and DRW samples, enterococcus counts were higher in winter (2.23 and 3.0 log cfu·100 mL^−1^, respectively) and in fall (1.89 and 2.45 log cfu·100 mL^−1^, respectively). Significant differences (*p* < 0.05) in bacterial counts were observed between the sampling sites. Enterococcus counts did not differ significantly across seasons (Figure 1).

### 3.3. Analysis of Correlations between Enterococci, Vancomycin Resistance Genes, Virulence Factors, Physicochemical Parameters, Sampling Site, and Season

The correlation analysis did not reveal significant differences (*p* < 0.05) between any of the examined parameters (excluding temperature) and season. Therefore, microbial parameters determined in different seasons are not presented. A negative correlation (r = −0.886) was noted between enterococcus counts and sampling site, whereas significant positive (r = 0.792) and negative (r = −0.606) correlations were determined between bacterial counts vs. water pH. Sampling sites were also significantly correlated with selected enterococcus species (*E. faecalis*, *E. faecium*, *E. avium*) (r = −0.599–0.620), resistance to the analyzed antibiotic groups (excluding TEC, VAN and DO), *VC2/VC3* genes (r = −0.497), virulence factors (as, cpd, ccf), and water pH (r = −0.703), (Table S4A, Supplementary Materials). Enterococcus species were correlated with resistance to antibiotics, selected resistance genes (mainly *van*B and *van*C1) and virulence factors. *Enterococcus faecalis* and *E. faecium* were correlated with all examined virulence factors (r = 0.482–0.906) (excluding *E. faecium* and COB). Significant correlations were also noted between drug-resistant strains, virulent strains, and strains harboring *van* genes, subject to the analyzed factor (Table S4B,D, Supplementary Materials).

### 3.4. Species Diversity, Percentage Composition and Multidrug Resistance of Enterococci in Samples of River Water and Wastewater

Seven Enterococcus species were identified in the total number of 283 strains isolated from water and wastewater samples: *E. faecalis*, *E. faecium*, *E. durans*, *E. avium*, *E. hirae*, *E. gallinarum*, and *E. casseliflavus/flavescens*. Strains not identified to species level were labeled as other *Enterococcus* spp. *Enterococcus faecium* was the dominant species in wastewater samples regardless of the sampling site, and its percentage share ranged from 38.8% to 42.9%. The second most abundant species was *E. faecalis* which represented 29.4% to 31.0% of the species identified in wastewater samples. The abundance of the remaining species in wastewater was much lower and did not exceed several percent. *Enterococcus gallinarum* (31.3%) and *E. faecium* (25%) were dominant in DRW samples, and *E. faecalis* (29.4%) was most abundant in URW samples. *Enterococci* spp. accounted for several to more than 10% of all bacterial species in wastewater samples and more than 70% in DRW samples (Table 2).

The isolated strains were tested for resistance to 13 antimicrobials belonging to 10 drug groups. The results were used to classify the bacterial strains as MDR and XDR. Two hundred and thirty-five isolates representing 94% of all enterococci identified in wastewater and 23 isolates representing 69.7% of all strains identified in river water were classified as MDR. Samples of HWW contained 79 MDR strains (94.0%) and 4 XDR strains (4.8%). In UWW and TWW samples, 67 (98.5%) and 91 (92.9%) strains were classified as MDR, respectively, and 1 strain in each sample (1.5% and 1.0%) was classified as XDR. Multidrug-resistant *E. faecium* was predominant in HWW (34 strains, 94.4%) and UWW (27 strains, 96.4%) samples, and *E. faecalis* was most abundant in TWW samples (28 strains, 96.6%). The remaining species isolated from wastewater samples were classified as MDR (Table 2).

**Figure 2:**
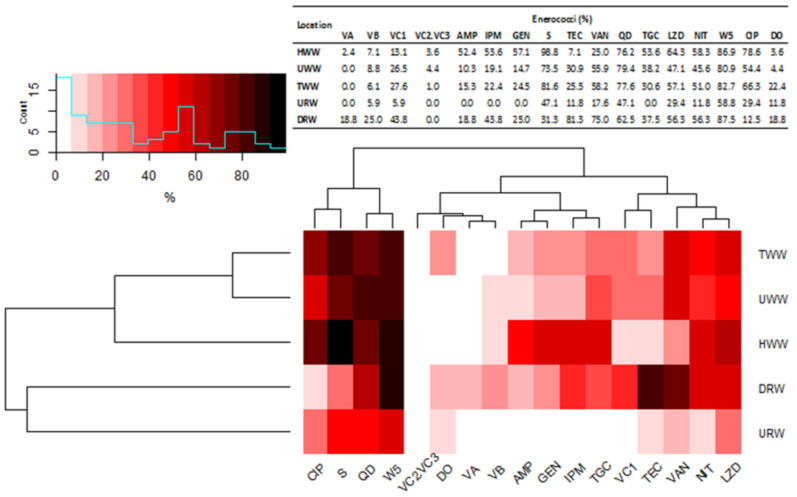
Heatmap with percentage content of studied multidrug resistant enterococcus among wastewater and river water (HWW—hospital wastewater; UWW—untreated wastewater; TWW—treated wastewater URW—upstream river water; DRW—downstream river).

Three *E. faecalis* strains in URW samples (60.0%) and four *E. faecium* strains in DRW samples (100%) were classified as MDR. Most of the strains (50–83.0%) isolated from wastewater samples were resistant to streptomycin (S) and trimethoprim (W5). More than 78% of *E. faecium* and E. faecalis isolates were resistant to these antibiotics. The percentage of isolates resistant to low concentrations of vancomycin ranged from 22% (*E. faecium*) in HWW to 70% (*E. faecalis*) in UWW samples. Most of the identified isolates were sensitive to doxycycline (DO). Bacterial species in DRW and URW samples had similar antibiotic resistance profiles to the strains isolated from wastewater. Most strains identified in URW samples were resistant to one, three, or six antibiotics, whereas most strains isolated from DRW samples were resistant to ten antimicrobials (Figure 2). *Enterococcus faecium* and *E. faecalis*, which cause most human and animal infections, were resistant mainly to teicoplanin (TEC), trimethoprim (W5), and streptomycin (S). None of the strains isolated from URW samples were resistant to ampicillin (AMP), imipenem (IMP), gentamicin (GEN), or tigecycline (TGC) (Table S5 in Supplementary Materials).

### 3.5. Identification of Vancomycin Resistance Genes (Van) in Enterococci Isolated from Wastewater and River Water

**Figure 3:**
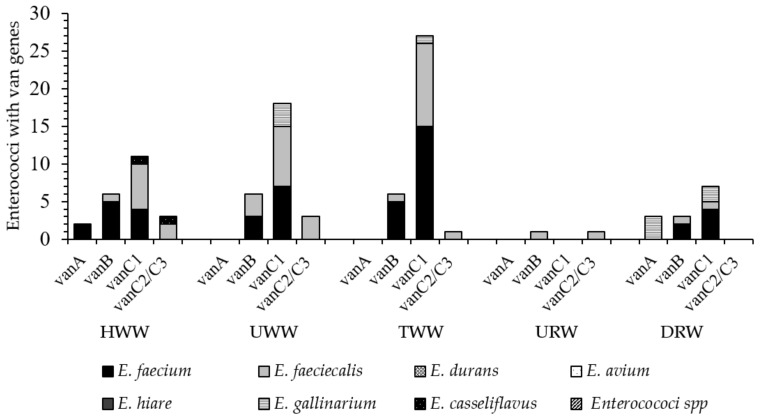
The number of enterococci with van genes in wastewater and river water (HWW—hospital wastewater; UWW—untreated wastewater; TWW—treated wastewater URW—upstream river water; DRW—downstream river).

Out of the 250 strains isolated from wastewater, only the strains identified in HWW samples harbored all vancomycin resistance genes. The number of vancomycin-resistant enterococci in HWW samples ranged from 2 strains (2.4%) with *van*A genes to 15 strains (39.5%) with *van*C1 genes. Six strains harboring *van*B genes were identified in UWW and TWW samples each (8.8% and 6.1%, respectively). The presence of the *van*C1 genes was noted in 18 (26.5%) and 27 (27.6%) of UWW and TWW isolates, respectively. One strain (1.02%) in TWW samples and three strains (4.41%) in UWW samples, mostly *E. faecium* and *E. faecalis*, harbored *van*C2/C3 genes. The predominant VRE species were *E. faecium* (*van* B genes, 5 strains, 13.8%) in HWW and *E. faecalis* (*van* C genes, 8 strains, 40%) in UWW samples. Five strains (13.1%) with *van*B genes and 15 strains (39.5%) with *van*C1 were isolated from TWW samples. All *E. gallinarum* and *E. casseliflavus* strains identified in wastewater harbored *van*C1 and *van*C2/C3 genes that confer natural resistance to vancomycin. Vancomycin-resistance genes were not identified in the remaining species isolated from the wastewater (Figure 3).

In URW samples, only one *E. faecalis* (5.88%) strain with phenotypic resistance to vancomycin harbored *van* B and *van* C2/C3 genes. The percentage of VRE strains was much higher in DRW samples, and it ranged from 18.7% (3 *E. galinarium* strains with *van* A genes) to 43.75% (1 *E. faecalis* strains, 4 *E. faecium* strains and 2 *E. gallinarum* strains with *van* C1 genes) Figure 2.

### 3.6. Identification of Genes Encoding Virulence Factors in Enterococci Isolated from Wastewater and River Water

**Figure 4:**
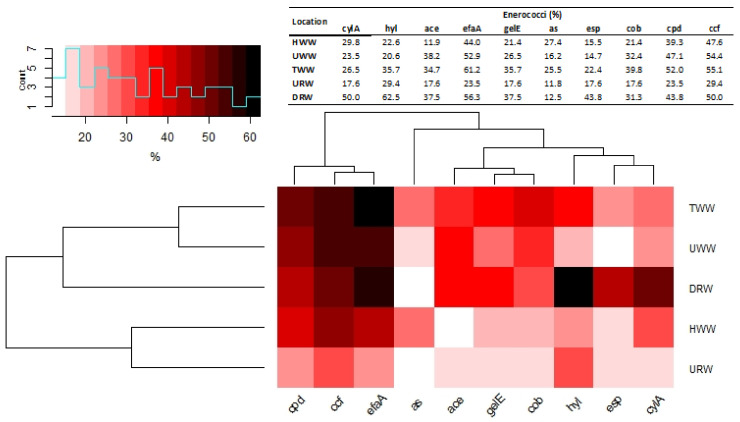
Heatmap with percentage content of studied virulence enterococcus among wastewater and river water (HWW—hospital wastewater; UWW—untreated wastewater; TWW—treated wastewater URW—upstream river water; DRW—downstream river).

Genes encoding virulence factors were detected in 188 strains (75.2%) in wastewater and in 22 strains (66.6%) in river water samples. Strains with the highest number of virulence factors were detected mainly in TWW samples, and strains with the smallest number of virulence factors were determined in URW samples. Most virulence factors were associated with *efa*A and *ccf* genes. These genes were most abundant in the strains isolated from TWW samples (61.2% and 55.1%, respectively), followed by the strains isolated from UWW (52.9% and 54.4%) and HWW (44.0% and 47.6%) samples. The parentage of selected virulence genes (*hyl*, *ace*, *efa*A, *gel*E, *as*, *esp*) was higher in TWW (several to around 10%) than in HWW and UWW. In URW samples, 23.5% and 29.4% of the strains harbored *efa*A and *ccf* genes, respectively, but their percentage was lower than in wastewater isolates. The smallest number of strains in HWW (12.0%) harbored *ace* genes, and the smallest number of strains in UWW (14.7%) and TWW (22.4%) samples harbored *esp* genes. Strains with *as* genes were least frequently isolated from URW (11.8%) and DRW (12.5%) samples. (Figure 4). In TWW samples, the percentage of *E. faecalis* harboring various virulence genes ranged from 26.9% to 100% (Table S6, Supplementary Materials). Virulent *E. faecium* strains accounted for 0% (HWW) to 55.3% (TWW) of the isolates. More than 70% of *E. faecalis* strains and 40–60% of *E. faecium* strains isolated from UWW and TWW samples harbored *efaA*, *cpd,* and *ccf* genes. *Enterococcus faecium* strains with *cylA*, *hyl,* and *ace* genes were predominant (more than 20%) only in HWW samples on average, 10 virulence genes were identified in *E. faecium* and *E. faecalis*. In the remaining enterococci, the number of virulence genes was around 50% lower on average (Table S6, Supplementary Materials).

The presence of correlations between the abundance of vancomycin resistance genes (*van*) and phenotypic resistance to antibiotics in strains isolated from wastewater and river water samples was determined in a correlation analysis using the Ward’s method, and it revealed three main clusters (Figure 3). The first cluster was composed of strains resistant to CIP, S, QD, and W5. The second cluster comprised strains harboring *van*C2/C3, *van*A, and *van*B genes, as well as strains resistant to AMP, GEN, IPM, and TGC. The third cluster grouped strains with *van*C1 genes which were resistant to TEC, VAN, NIT, and LZD. The strains harboring *van* genes and MDR strains were divided into two main clusters based on their source. The first cluster was composed of strains isolated from TWW, UWW, and HWW samples, and the second cluster comprised strains isolated from DRW and URW samples.

Two clusters were identified based on the results of the virulence analysis. The first cluster grouped strains with *cpd*, *ccf,* and *efaA* genes that were most abundant in TWW, UWW, and DRW samples. The second cluster was subdivided into two groups composed of strains with *ace*, *gel*E, and *cob* genes (that were also most abundant in TWW, UWW, and DRW samples) and strains harboring *hyl*, *esp,* and *cyl*A, which were most prevalent in DRW samples. Strains harboring *as* genes were isolated from both subgroups, and they were detected infrequently, mostly in HWW and TWW samples (Figure 4).

## 4. Discussion

### 4.1. Enterococcus Counts in Wastewater and River Water Samples

Rapid economic growth and industrialization contribute to growing levels of anthropogenic pressure on water resources around the world. Biological micropollutants that reach water bodies with treated wastewater are a serious and often marginalized problem. These pathogens are often correlated with the incidence of gastrointestinal diseases, the presence of ARGs and ARB, and they pose a significant epidemiological risk. Despite the above, there are no detailed guidelines for disinfecting treated wastewater or controlling microbiological pollution in water bodies (Council Directive 91/271/EEC; OJ, item 1800), [48]. This study analyzed the pool of virulent and antibiotic-resistant enterococci in municipal wastewater (including hospital wastewater) before and after mechanical and biological treatment in WWTP and in river water sampled downstream and upstream from the discharge point. Significant differences (*p* < 0.05) in enterococcus counts were noted between sampling sites, but not between seasons. The average enterococcus counts in UWW and HWW samples were determined at 6 log cfu·100 mL^−1^. Wastewater treatment reduced the abundance of enterococci by around 97%, but TWW samples contained more than several dozen thousand bacterial cells per 100 mL. The analyzed WWTP has a daily processing capacity of around 32,000 m^3^, which indicates that millions of bacterial cells reach the river each day. Enterococcus counts were more than two-fold lower in the samples collected upstream from the effluent discharge point, which suggests that the evacuation of treated wastewater compromises the microbiological quality of river water. The abundance of indicator bacteria, including enterococci, as well as ARGs and ARB is higher in water bodies that act as receptacles of municipal wastewater in urban areas [5,6,49,50,51]. An increase in enterococcus counts points to the contamination of river water with human and animal feces. Fecal contamination is also associated with higher abundance of potentially pathogenic bacteria such as *Aeromonas hydrophila*, *Listeria monocytogenes*, *Salmonella* spp., *Pseudomonas aeruginosa*, *Campylobacter* spp., *Vibrio* spp., and *Yersinia* spp. [52,53,54,55,56]. Human activities (wastewater treatment, agriculture, tourism) also contribute to the spread of MDR enterococci and VRE both in and outside the hospital environment around the world [14,35,36,57].

### 4.2. Species Composition, Multidrug Resistance and the Presence of Van Genes in Enterococci Isolated from Water and Wastewater Samples

In the group of seven identified enterococcus species, *E. faecium* (102 strains, 40.8%), *E. faecalis* (75 strains, 30.0%) were most abundant in all wastewater and river water samples, excluding DRW samples where *E. gallinarium* was most prevalent (29.4%). The percentage of the remaining enterococcus species (*E. durans*, *E. avium*, *E. hirae*, *E. casseliflavus/flavescens*) did not exceed 8% in the analyzed samples. Higher counts and percentage of *E. faecium* and *E. faecalis* in samples of untreated and treated wastewater and in samples of river water collected in the vicinity of the discharge point were also reported in other studies [49,58,59,60]. *Enterococcus faecalis* and *E. faecium* are the most abundant species in human and animal feces, wastewater and water contaminated with fecal matter [15,36,61,62]. In turn, *E. hirae* was most prevalent in wastewater in Portugal and the USA [63,64], whereas *E. durans* (24%) was the second most prevalent species after *E. faecalis* in treated and untreated hospital waste in Eastern Cape in the Republic of South Africa [65]. The composition of wastewater microbiota and their antibiotic resistance profiles can differ depending on the type of wastewater and the applied treatment methods [50,66]. A pilot study investigating the effectiveness of UV radiation in wastewater treatment demonstrated that UV light decreased enterococcus counts in treated wastewater by 3.1–3.3 log CFU relative to untreated wastewater. Ultraviolet radiation decreased the abundance of enterococci in treated wastewater to a level that is generally noted in unpolluted waters [8,12]. Despite the fact that treated wastewater contributes to environmental pollution, it does not have to be disinfected before it is evacuated to surface water bodies in Poland [67].

Multidrug-resistant enterococci accounted for 94% and 69.7% of the strains isolated from wastewater and river water samples, respectively. More than 87% of *E. faecium* and *E faecalis* strains isolated from wastewater, and all *E. faecium* and *E faecalis* strains isolated from DRW were classified as MDR. Multidrug-resistant strains represented 60% of the isolates from URW samples. Individual strains of the remaining enterococcus species (*E. durans*, *E. avium*, *E. hirae*, *E. gallinarum,* and *E casseliflavus/flavescens*) were also resistant to multiple antimicrobials. In wastewater samples, up to 5.6% of *E. faecium* and *E. faecalis* were XDR. These strains are particularly dangerous because they are resistant to at least one antibiotic in all but two antimicrobial groups [45]. Multidrug-resistant enterococci are responsible for a high percentage of hospital infections that are difficult to diagnose and treat [8,63]. Most of the isolated MDR strains were resistant to 7–8 of the tested antibiotics. More than 80% of those strains were resistant to streptomycin (aminoglycosides) and trimethoprim (chemotherapeutics), whereas the smallest number of strains (up to 22%) were resistant to doxycycline (tetracyclines). *Enterococcus faecalis* and *E. faecium* are highly resistant to aminoglycosides around the world [12,68], including in Poland [14]. More than 50% of *E. faecalis* and *E. faecium* strains identified in Ethiopia were resistant to vancomycin, penicillin, amoxicillin, doxycycline, and tetracycline, and 60.0% of all identified enterococci were classified as MDR [31]. In the present study, enterococcus strains isolated 500 m downstream from the wastewater discharge point had similar antibiotic resistance profiles, and most of them were resistant to trimethoprim (87.5%), vancomycin, and teicoplanin (more than 75% of the strains). These results clearly indicate that antibiotic-resistant enterococci are transferred to rivers with TWW. Numerous studies have demonstrated that the percentage of MDR enterococci and other bacterial groups continues to increase in TWW evacuated to water bodies [33,36,37,41,57,58].

Various *van* genes were identified in enterococcus strains characterized by phenotypic resistance to vancomycin and isolated from river water and wastewater. The cluster analysis involving Ward’s method revealed two groups of enterococcus strains whose antibiotic resistance profiles were most highly correlated with *van* genes. The first group comprised the strains isolated from UWW, TWW, and HWW samples, and the second group consisted of strains isolated from DRW and URW samples. Strains harboring all of the examined resistance genes (*van*A, *van*B, *van*C1, and *van*C2/C3) were identified mainly in HWW samples, but their percentage was generally low in the range of 2.4% (*van*A) to 13.1% (*van*C1). The above genes were detected in 1.02% (*van*C2/C3) to 27.6% (*van*C1) of the strains isolated from UWW and TWW samples. *van*C1 and *van*C2/3 genes conditioning natural resistance to vancomycin were most abundant in *E. gallinarum* and *E. casseliflavus*. The abundance of *van* genes was highest in *E. faecalis* and *E. faecium* isolates. These strains (20–70%) were also resistant to vancomycin in the disc diffusion test, and they included isolates resistant to high concentrations of vancomycin (MIC ≥ 32 to ≥ 256 mg·L^–1^; data not shown), which can be classified as VRE. The proportion of VRE strains exceeded 10%, and it was somewhat higher in TWW than UWW samples. Individual VRE strains were also noted in DRW samples. A similar percentage of VRE strains (27.0%) was reported in a WWTP in the United States [69]. In other studies, the proportions of VRE strains in wastewater ranged from 2% to 52%, depending on the treatment method, type of wastewater, and treatment stage [69,70,71]. Enterococci resistant to high concentrations of vancomycin (20 mg·L^–1^) were isolated from surface water (7%) in many European countries (Sweden, Spain, United Kingdom). These bacteria were also identified in UWW (71.0%) and TWW (36.0%). Resistance to vancomycin was determined mainly in *E. faecalis* strains harboring *van*A genes [60]. In the current study, *van*B, *van*C1, and *van*C2/C3 were the main genetic determinants of resistance to vancomycin. The presence of *van*C1 genes in *E. faecium* and *E. faecalis* was surprising. Until recently, it was believed that *van*C1 is encoded chromosomally and is not horizontally transferred. However, this gene is increasingly often identified in *E. faecium* and *E. faecalis*, which suggests that it can be acquired from *E. gallinarum* and *E. casseliflavus* by horizontal transfer [72]. Multidrug-resistant enterococci and VRE have been spreading at an alarming rate in both hospitals [31,73] and the natural environment [41,65]. These pathogens could originate from various sources which have not been fully elucidated to date. Enterococci harboring virulence factors that cause human infections and spread in the environment also pose a considerable problem [35,74].

### 4.3. Characteristics of Virulent Enterococci in Wastewater and River Water Samples

In wastewater samples, 75.2% of the isolated enterococcus strains harbored virulence factors (*cylA, hyl, ace, efaA, as, gelE, esp, cob, cpd, ccf*). The cluster analysis involving Ward’s method revealed the strongest correlations between sampling sites and the prevalence of strains with virulence genes in untreated (UWW) and treated (TWW) municipal wastewater as well as in river water sampled downstream from the wastewater discharge point (DRW). These samples were characterized by the highest percentage of strains containing sex pheromones *cpd* and *ccf* (21.4–55.1%) and cell wall adhesin *efaA* (44.1–61.2%). Sex pheromones participate in the exchange of genetic material and, consequently, induction of virulence. They also colonize hosts and cause inflammations [75,76]. Enterococcus strains with dominant sex pheromone genes were also identified in the South Nation River watershed in Ontario, Canada [65]. In the present study, 11.9% to 38.2% of the strains isolated from wastewater also harbored genes encoding cytolysin (*cyl*A), collagen-binding surface protein (*ace*) (UWW) and gelatinase (*gel*E). Virulence genes are often associated with hospital infections caused by pathogenic enterococci [35,77]. In this study, *E. faecalis* (5.0–100%) and *E. faecium* (0–57.1%) were characterized by the highest number and diversity of virulence genes, with a predominance of *efaA, cpd,* and *ccf*. Similar observations were made in hospital strains in Bulgaria [36]. Virulence genes *asa* (aggregation substance) and *cyl*A (cytolytic toxin) are often predominant in enterococci isolated from hospitals, the natural environment, animals, and wastewater [35,78]. Enterococci isolated from the Ganges River in India were characterized by a similar virulence profile [12]. In the Łyna River, 87% of the strains isolated downstream from the effluent discharge point harbored different virulence genes, but only 47.1% of such strains were isolated upstream from the discharge point. The isolated strains had similar virulence profiles to the strains identified in UWW and TWW samples. However, the highest percentage of these strains (62.5%) harbored genes encoding hyaluronidase (*hyl*) which contributes to connective tissue damage and facilitates the spread of bacteria in the host organism [18]. These genes were most abundant in vancomycin-resistant strains that harbored *van* genes and were isolated from URW samples. *Hyl* genes are frequently identified in clinical VRE isolates, but they are also present in vancomycin-susceptible enterococci (VSE) [79]. Most *E. faecalis* and *E. faecium* strains containing virulence genes, including *esp*, *gel*E, *cyl*A and *hyl*, are associated with human infections [35,77]. The spread of MDR enterococci and VRE in the environment is particularly dangerous because these pathogens are not easy to detect or treat. These strains cause life-threatening infections and pose a significant epidemiological risk.

## 5. Conclusions

The results of this study indicate that WWTPs are significant sources of MDR and virulent enterococci in the environment. The strains isolated from TWW samples were characterized by higher levels of antibiotic resistance and virulence than those isolated from UWW and, in many cases, HWW samples. This observation indicates that bacteria undergo selective pressure during wastewater treatment and are transformed into MDR strains by accumulating genes that encode resistance to various antimicrobials. The enterococci isolated from DRW and wastewater were characterized by similar multidrug resistance and virulence profiles, and significant correlations were observed between these groups of isolates. The counts of these bacteria were two-fold lower in river water sampled upstream from the effluent discharge point. These findings suggest that large numbers of VRE and MDR enterococci are released to water bodies even from WWTPs equipped with highly effective treatment systems. The spread of these bacteria along the river continuum poses a significant epidemiological risk and a threat to public health. Therefore, enterococcus emissions should be monitored, in the hospital environment, treated wastewater should be disinfected before it is evacuated to surface water bodies and that new wastewater treatment technologies should be developed to minimize the risk of exposure to these pathogens.

## Figures and Tables

**Table 1:** Physicochemical properties of wastewater and river water.

Sampling Sites	Parameters	
	pH	Temperature (°C)
HWW	8.35 ± 0.62 ^a^	14.37 ± 6.96
	(7.60−9.20) ^b^	(7.58−25.89)
UWW	8.39 ± 0.42	14.74 ± 6.60
	(8.02−9.22)	(7.58−25.85)
TWW	8.40 ± 0.35	15.55 ± 8.67
	(8.02−9.01)	(7.47−26.80)
URW	7.16 ± 0.41	11.76 ± 4.87
	(6.40−7.50)	(0.0−21.10)
DRW	7.21 ± 0.65	14.31 ± 6.0
	(6.50−8.40)	(9.30−24.70)
*p* value ^c^	0.0011 *	0.8452

^a^ mean and standard deviation (±SD); ^b^ range ^c^ Kruskal–Wallis test, statistically significant differences [* *p* ≤ 0.001] between sampling stations. HWW—hospital waste water; UWW and TWW—untreated and treated wastewater; URW and DRW—upstream and downstream river water.

**Table 2:** Number and percentage of multidrug-resistant (MDR) and extensively drug-resistant (XDR) enterococci in wastewater and river water.

	Sewage			River	
Species	HWW	UWW	TWW	URW	DRW
	Strains: number (%)				
	MDR (%)				
	XDR (%)				
*E. faecium*	36 (42.9)	28 (41.2)	38 (38.8)	0	4 (25.0)
	34 (94.4)	27 (96.4)	33 (86.8)	0	4 (100)
	2 (5.6)	1 (3.57)	1 (2.6)	0	0
*E. faecalis*	26 (31.0)	20 (29.4)	29 (29.6)	5 (29.4)	1 (6.3)
	23 (88.5)	19 (95.0)	28 (96.6)	3 (60)	1 (100)
	1(3.8)	0	0	0	0
*E. durans*	1 (1.2)	0	3 (3.1)	0	0
	1 (100)	0	3 (100)	0	0
*E. avium*	7 (8.3)	4 (5.9)	2 (2.0)	0	0
	7 (100)	4 (100)	2 100)	0	0
*E. hirae*	2 (2.4)	1 (1.5)	4 (4.1)	0	0
	2 (100)	1 (100)	4 (100)	0	0
*E. gallinarum*	1 (1.2)	3 (4.4)	1 (1.0)	0	5 (31.3)
	1 (100)	3 (100)	1 (100)	0	4 (80)
*E. casseliflavus/*	1 (1.2)	0	0	0	0
*flawescens*	1 (100)	0	0	0	0
Other	10 (11.9)	12 (17.6)	21 (21.4)	12 (70.6)	6 (37.5)
*Enterococcus* spp.	10 (100)	13 (100)	20 (95.2)	5 (41.6)	6 (100)
	1 (10.0)	0	0	0	0
Number of strains	84	68	98	17	16
MDR (%)	79 (94.0)	67 (98.5)	91 (92.9)	8 (47.0)	15 (93.8)
XDR (%)	4 (4.8)	1(1.5)	1(1.0)	0	0

## Data Availability

Data is contained within the article or Supplementary Materials.

## Supplementary Materials

The following are available online at https://www.mdpi.com/1660-4601/18/2/563/s1. Figure S1: Technological scheme of wastewater treatment plant. Table S1: Oligonucleotide sequences and product size of primers for identifying enterococci. Table S2: Sequences of oligonucleotides and primers for identifying vancomycin resistance genes in enterococci. Table S3: Sequences of oligonucleotides and primers for identifying virulence factors in enterococci. Table S4: (Part A–B) Correlations between the analyzed physicochemical parameters, total enterococcus counts, different species, and the number of MDR and virulent strains (Spearman’s rank correlation coefficient; marked in red are values with *p* < 0.05). Table S5: Number and percentage of antibiotic resistant enterococci in wastewater and river water. Table S6: Number and percentage of virulent enterococci in wastewater and river water.
