# Supplementary material for: The Prevalence of Virulent and Multidrug-Resistant Enterococci in River Water and in Treated and Untreated Municipal and Hospital Wastewater
Source: Int J Environ Res Public Health. 2021 Jan 11;18(2):563. doi: 10.3390/ijerph18020563 (PMC7827636; doi:10.3390/ijerph18020563)
Supplement: Supplementary file 1 [file ijerph-18-00563-s001.pdf]

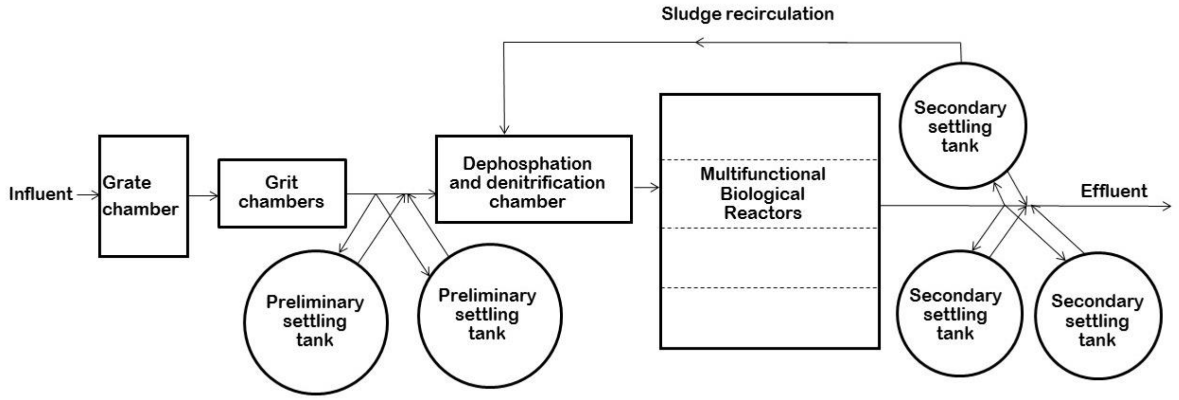


**Figure S1**. Technological scheme of Wastewater Treatment Plant

**Table S1.** Oligonucleotide sequences and product size of primers for identifying enterococci.

| Name | Gene | Sequences (5'-3') | Lenght (bp) | References |
|---|---|---|---|---|
| *Enterococcus* | *tuf* | TACTGACAAACCATTCATGATG<br>AACTTCGTCACCAACGCGAAC | 112 | Ke et al., [1] |
| *Enterococcus* | *sod*A | TCA ACC GGG GAG GGT<br>ATT ACT AGC GAT TCC GG | 733 | Deasy et al., [2] |
| *E. faecalis* | *ddl* | ATCAAGTACAGTTAGTCTTTATTAG<br>ACGATTCAAAGCTAACTGAATCAGT | 941 | Kariyama et al., [3] |
| *E. faecium* | *ddl* | TTGAGGCAGACCAGATTGACG<br>TATGACAGCGACTCCGATTCC | 658 | Cheng et al., [4] |
| *E. durans* | *ddl* | CCTACTGATATTAAGACAGCG<br>TAATCCTAAGATAGGTGTTTG | 295 | Jackson et al., [5] |
| *E. hirae* | *ddl* | CTTTCTGATATGGATGCTGTC<br>TAAATTCTTCCTTAAATGTTG | 187 | |
| *E. avium* | *ddl* | GCTGCGATTGAAAAATATCCG<br>AAGCCAATGATCGGTGTTTTT | 368 | |
| *E. gallinarum* | *van*C1 | GGTATCAAGGAAACCTC<br>CTTCCGCCATCATAGCT | 822 | Dutka–Malen et al., [6] |
| *E. casseliflavus* | *van*C2/C3 | CGGGGAAGATGGCAGTAT<br>CGCAGGGACGGTGATTTT | 484 | Kariyama et al., [3] |
| Control | *rrs*(16S rRNA) | GGATTAGATACCCTGGTAGTCC<br>TCGTTGCGGGACTTAACCCAAC | 320 | Van de Klundert and Vliegenthart et al., [7] |

**Table S2.** Sequences of oligonucleotides and primers for identifying vancomycin resistance genes in enterococci.

| Gene | Sequences (5'-3') | Lenght (bp) | References |
|---|---|---|---|
| *van*A | CATGAATAGAATAAAAGTTGCAATA<br>CCCCTTTAACGCTAATACGATCAA | 1.030 | Kariyama et al., [3] |
| *van*B | GTGACAAACCGGAGGCGAGGA<br>CCGCCATCCTCCTGCAAAAAA | 433 | |
| *van*C1 | GGTATCAAGGAAACCTC<br>CTTCCGCCATCATAGCT | 822 | |
| *van*C2/C3 | CGGGGAAGATGGCAGTAT<br>CGCAGGGACGGTGATTTT | 484 | |

**Table S3**. Sequences of oligonucleotides and primers for identifying virulence factors in enterococci

| Virulence factor | Gene | Sequences (5'-3') | Lenght (bp) | References |
|---|---|---|---|---|
| Cytolysin | *cyl*A | Cyl1–ACTCGGGGATTGATAGGC<br>Cyl2–GCTGCTAAAGCTGCGCTT | 688 | Vankerckhoven et al., [8] |
| Hyaluronidase | *hyl* | Hyl1–ACAGAAGAGCTGCAGGAAATG<br>Hyl2–GACTGACGTCCAAGTTTCCAA | 276 | |
| Adhesion colaagen | *ace* | Ace1–AAAGTAGAATTAGATCCACAC<br>Ace2–TCTATCACATTCGGTTGCG | 320 | Mannu et al., [9] |
| Antigen A | *efa*A | efaA1–CGTGAGAAAGAAATGGAGGA<br>efaA2–CTACTAACACGTCACGAATG | 499 | |
| Gelatinase | *gel*E | gelE1–AGTTCATGTCTATTTTCTTCAC<br>gelE2–CTTCATTATTTACACGTTTG | 402 | |
| Aggregation substance | *as* | AS1–CCAGTAATCAGTCCAGAAACAACC<br>AS2–TAGCTTTTTTCATTCTTGTGTTTGTT | 406 | |
| Enterococcal surface protein | *esp* | Esp1–TTACCAAGATGGTTCTGTAGGCAC<br>Esp2–CCAAGTATACTTAGCATCTTTTGG | 913 | Shankar et al., [10] |
| Sex pheromones | *cpd* | Cpd1–TGGTGGGTTATTTTTCAATTC<br>Cpd2–TACGGCTCTGGCTTACTA | 782 | Eaton and Gasson [11] |
| Sex pheromones | *cob* | Cob1–AACATTCAGCAAACAAAGC<br>Cob2–TTGTCATAAAGAGTGGTCAT | 1405 | |
| Sex pheromones | *ccf* | Ccf1–GGGAATTGAGTAGTGAAGAAG<br>Ccf2–AGCCGCTAAAATCGGTAAAAT | 543 | |

**Table S4**–Part A. Correlations between the analyzed physicochemical parameters, total enterococcus counts, different species and the number of MDR and virulent strains (Spearman's rank correlation coefficient; marked in red are values with $p < 0.05$).

| Variable | Season | Sampling sites | Temperature | pH | Dissolved oxygen ($mg\ O_2\ L^{-1}$) | Enterococci ($log\ cfu \cdot 100mL^{-1}$) |
|---|---|---|---|---|---|---|
| Sampling sites | 0.000 | | | | | |
| Temperature | 0.374 | -0.086 | | | | |
| pH | 0.337 | -0.703 | 0.530 | | | |
| Dissolved oxygen ($mg\ O_2\ L^{-1}$) | -0.249 | 0.632 | -0.200 | -0.580 | | |
| Enterococci ($log\ cfu \cdot 100mL^{-1}$) | 0.064 | -0.886 | 0.162 | 0.792 | -0.606 | |
| Other *Enterococcus* spp | 0.035 | -0.146 | -0.023 | 0.220 | 0.294 | 0.199 |
| *E. faecalis* | -0.304 | -0.613 | -0.183 | 0.466 | -0.204 | 0.530 |
| *E. faecium* | 0.179 | -0.620 | 0.058 | 0.657 | -0.495 | 0.633 |
| *E. durans* | -0.088 | -0.139 | 0.047 | 0.268 | -0.007 | 0.139 |
| *E. avium* | -0.147 | -0.599 | -0.006 | 0.269 | -0.123 | 0.532 |
| *E. hiare* | -0.163 | -0.184 | -0.209 | 0.185 | -0.043 | 0.179 |
| *E. gallinarum* | 0.046 | 0.181 | -0.050 | -0.084 | -0.245 | -0.063 |
| *E. casseliflavus/flavescens* | 0.308 | -0.324 | -0.118 | 0.227 | -0.575 | 0.232 |
| AMP | 0.210 | -0.680 | -0.015 | 0.456 | -0.581 | 0.506 |
| IPM | 0.158 | -0.715 | 0.046 | 0.569 | -0.541 | 0.576 |
| GEN | 0.095 | -0.681 | 0.096 | 0.572 | -0.496 | 0.534 |
| S | -0.023 | -0.693 | -0.046 | 0.605 | -0.317 | 0.619 |
| TEC | -0.021 | -0.034 | 0.054 | 0.235 | 0.006 | 0.229 |
| VAN | -0.237 | -0.292 | -0.105 | 0.347 | 0.129 | 0.397 |
| QD | -0.069 | -0.588 | -0.103 | 0.572 | -0.286 | 0.582 |
| TGC | 0.282 | -0.678 | -0.085 | 0.631 | -0.507 | 0.673 |
| LZD | 0.208 | -0.518 | -0.094 | 0.550 | -0.295 | 0.509 |

| | | | | | | |
|---|---|---|---|---|---|---|
| NIT | 0.344 | -0.554 | 0.148 | 0.713 | -0.388 | 0.591 |
| W5 | 0.168 | -0.639 | 0.014 | 0.670 | -0.354 | 0.646 |
| CIP | 0.096 | -0.671 | -0.003 | 0.671 | -0.377 | 0.633 |
| DO | -0.129 | -0.012 | -0.205 | 0.092 | 0.381 | 0.053 |
| *van*A | 0.088 | 0.092 | -0.234 | -0.324 | -0.049 | -0.131 |
| *van*B | -0.198 | -0.256 | -0.201 | 0.021 | -0.136 | 0.198 |
| *van*C1 | -0.178 | -0.259 | 0.162 | 0.350 | -0.046 | 0.331 |
| *van*C2/VC3 | 0.140 | -0.497 | 0.343 | 0.555 | -0.600 | 0.527 |
| *cyl*A | -0.081 | -0.428 | -0.099 | 0.320 | -0.400 | 0.365 |
| *hyl* | 0.044 | -0.267 | 0.233 | 0.486 | 0.051 | 0.304 |
| *ace* | -0.342 | -0.262 | -0.047 | 0.337 | 0.058 | 0.338 |
| *efa*A | -0.342 | -0.436 | -0.183 | 0.381 | -0.130 | 0.422 |
| *gel*E | -0.422 | -0.306 | -0.037 | 0.278 | -0.043 | 0.254 |
| *as* | -0.227 | -0.556 | -0.019 | 0.396 | -0.237 | 0.446 |
| *esp* | -0.064 | -0.214 | -0.116 | 0.287 | -0.166 | 0.173 |
| *cob* | -0.430 | -0.291 | -0.135 | 0.162 | 0.210 | 0.273 |
| *cpd* | -0.356 | -0.459 | -0.088 | 0.374 | -0.032 | 0.441 |
| *ccf* | -0.347 | -0.512 | -0.135 | 0.372 | -0.108 | 0.474 |

AMP–ampicillin; IPM–imipenem; GEN–gentamicin; S–streptomycin; TEC–teicoplanin; VAN–vancomycin; QD–quinupristin/dalfopristin; TGC–tigecycline; LZD–linezolid; NIT–nitrofurantoin; W5–trimethoprim; CIP–ciprofloxacin; DO–doxycycline; vancomycin resistance genes (vanA-C2/C3); virulence factors: cytolysin (cylA), aggregation substance (AS), gelatinase (gel E), hyaluronidase (hyl), endocarditis antigen (efaA), factor encoding surface protein (esp), and sex pheromones (cob, cpd and ccf)

**Table S4**–Part B

| Variable | Other *Enterococcus* spp | *Enterococcus:* *faecalis* | *faecium* | *durans* | *avium* | *hiare* | *gallinarum* | *casseliflavus/ flavescens* |
|---|---|---|---|---|---|---|---|---|
| *E. faecalis* | 0.213 | | | | | | | |
| *E. faecium* | -0.001 | 0.668 | | | | | | |
| *E. durans* | 0.496 | 0.466 | 0.295 | | | | | |
| *E. avium* | 0.009 | 0.381 | 0.279 | -0.148 | | | | |
| *E. hiare* | 0.420 | 0.549 | 0.177 | 0.876 | 0.022 | | | |
| *E. gallinarum* | 0.066 | -0.007 | -0.020 | 0.101 | -0.121 | 0.133 | | |
| *E. casseliflavus/flavescens* | -0.243 | 0.242 | 0.512 | 0.360 | 0.076 | 0.393 | 0.118 | |
| AMP | -0.031 | 0.446 | 0.659 | 0.324 | 0.338 | 0.277 | -0.046 | 0.703 |
| IPM | 0.029 | 0.596 | 0.711 | 0.305 | 0.403 | 0.301 | -0.036 | 0.583 |
| GEN | 0.093 | 0.573 | 0.721 | 0.445 | 0.354 | 0.347 | -0.047 | 0.598 |
| S | 0.243 | 0.863 | 0.805 | 0.591 | 0.429 | 0.604 | -0.132 | 0.500 |
| TEC | 0.565 | 0.368 | 0.283 | 0.593 | -0.064 | 0.532 | 0.465 | -0.091 |
| VAN | 0.468 | 0.777 | 0.527 | 0.439 | 0.305 | 0.460 | 0.169 | -0.129 |
| QD | 0.231 | 0.890 | 0.760 | 0.581 | 0.406 | 0.675 | 0.006 | 0.473 |
| TGC | 0.018 | 0.629 | 0.807 | 0.240 | 0.423 | 0.334 | -0.092 | 0.636 |
| LZD | 0.069 | 0.712 | 0.826 | 0.395 | 0.338 | 0.450 | -0.091 | 0.614 |
| NIT | 0.107 | 0.613 | 0.845 | 0.397 | 0.273 | 0.366 | -0.091 | 0.580 |
| W5 | 0.268 | 0.790 | 0.885 | 0.504 | 0.390 | 0.496 | -0.026 | 0.501 |
| CIP | 0.231 | 0.772 | 0.819 | 0.534 | 0.314 | 0.530 | -0.142 | 0.581 |
| DO | 0.239 | 0.584 | 0.355 | 0.212 | 0.041 | 0.236 | -0.026 | -0.128 |
| *van*A | 0.025 | -0.074 | -0.031 | -0.128 | -0.077 | -0.119 | 0.435 | -0.075 |
| *van*B | -0.209 | 0.396 | 0.297 | -0.331 | 0.505 | -0.182 | 0.227 | -0.194 |
| *van*C1 | -0.007 | 0.537 | 0.581 | -0.044 | 0.450 | -0.115 | 0.221 | -0.135 |
| *van*C2/VC3 | 0.028 | 0.181 | 0.211 | -0.031 | 0.007 | -0.008 | 0.080 | 0.251 |
| *cyl*A | 0.127 | 0.625 | 0.755 | 0.490 | 0.210 | 0.384 | 0.211 | 0.478 |

| | | | | | | | | |
|---|---|---|---|---|---|---|---|---|
| *hyl* | 0.446 | 0.595 | 0.599 | 0.559 | 0.080 | 0.376 | 0.036 | -0.010 |
| *ace* | 0.260 | 0.737 | 0.560 | 0.291 | 0.240 | 0.257 | 0.110 | -0.214 |
| *efa*A | 0.343 | 0.886 | 0.633 | 0.591 | 0.335 | 0.635 | 0.140 | 0.185 |
| *gel*E | 0.299 | 0.724 | 0.524 | 0.418 | 0.143 | 0.304 | 0.154 | -0.150 |
| *as* | 0,134 | 0,813 | 0,770 | 0,465 | 0,422 | 0,351 | 0,087 | 0,343 |
| *esp* | -0,138 | 0,714 | 0,735 | 0,187 | 0,168 | 0,194 | 0,171 | 0,310 |
| *cob* | 0,206 | 0,766 | 0,482 | 0,244 | 0,469 | 0,252 | 0,023 | -0,183 |
| *cpd* | 0,259 | 0,886 | 0,644 | 0,395 | 0,506 | 0,404 | 0,142 | 0,063 |
| *ccf* | 0,256 | 0,906 | 0,664 | 0,420 | 0,511 | 0,461 | 0,128 | 0,133 |

**Table S4**–Part C

| Variable | AMP | IPM | GEN | S | TEC | VAN | QD | TGC | LZD | NIT | W5 | CIP | DO |
|---|---|---|---|---|---|---|---|---|---|---|---|---|---|
| IPM | 0.944 | | | | | | | | | | | | |
| GEN | 0.925 | 0.938 | | | | | | | | | | | |
| S | 0.730 | 0.818 | 0.817 | | | | | | | | | | |
| TEC | -0.005 | 0.097 | 0.133 | 0.360 | | | | | | | | | |
| VAN | 0.098 | 0.293 | 0.260 | 0.646 | 0.740 | | | | | | | | |
| QD | 0.549 | 0.681 | 0.654 | 0.949 | 0.450 | 0.740 | | | | | | | |
| TGC | 0.785 | 0.863 | 0.786 | 0.842 | 0.131 | 0.385 | 0.801 | | | | | | |
| LZD | 0.673 | 0.773 | 0.722 | 0.890 | 0.221 | 0.534 | 0.892 | 0.933 | | | | | |
| NIT | 0.678 | 0.783 | 0.770 | 0.846 | 0.255 | 0.463 | 0.819 | 0.923 | 0.952 | | | | |
| W5 | 0.667 | 0.776 | 0.770 | 0.948 | 0.459 | 0.682 | 0.930 | 0.885 | 0.924 | 0.920 | | | |
| CIP | 0.746 | 0.841 | 0.820 | 0.951 | 0.262 | 0.539 | 0.908 | 0.901 | 0.937 | 0.925 | 0.935 | | |
| DO | 0.004 | 0.193 | 0.076 | 0.439 | 0.305 | 0.737 | 0.527 | 0.298 | 0.529 | 0.406 | 0.444 | 0.447 | |
| *van*A | 0.211 | 0.200 | 0.069 | -0.071 | 0.347 | 0.124 | -0.138 | -0.003 | -0.092 | -0.130 | -0.020 | -0.139 | 0.061 |
| *van*B | 0.123 | 0.225 | 0.064 | 0.216 | 0.176 | 0.472 | 0.252 | 0.187 | 0.155 | 0.024 | 0.228 | 0.052 | 0.304 |
| *van*C1 | 0.003 | 0.167 | 0.163 | 0.372 | 0.334 | 0.691 | 0.462 | 0.236 | 0.354 | 0.329 | 0.467 | 0.288 | 0.491 |
| *van*C2/VC3 | 0.265 | 0.382 | 0.350 | 0.182 | -0.062 | -0.093 | 0.178 | 0.360 | 0.201 | 0.365 | 0.234 | 0.349 | -0.156 |
| *cyl*A | 0.597 | 0.592 | 0.656 | 0.714 | 0.506 | 0.520 | 0.662 | 0.531 | 0.556 | 0.517 | 0.733 | 0.611 | 0.119 |
| *hyl* | 0.316 | 0.471 | 0.559 | 0.675 | 0.658 | 0.724 | 0.630 | 0.450 | 0.580 | 0.655 | 0.723 | 0.614 | 0.520 |
| *ace* | -0.028 | 0.156 | 0.204 | 0.535 | 0.618 | 0.882 | 0.623 | 0.257 | 0.401 | 0.362 | 0.581 | 0.396 | 0.572 |
| *efa*A | 0.342 | 0.480 | 0.505 | 0.811 | 0.644 | 0.860 | 0.869 | 0.508 | 0.621 | 0.527 | 0.782 | 0.678 | 0.481 |
| *gel*E | 0.185 | 0.312 | 0.372 | 0.561 | 0.609 | 0.776 | 0.555 | 0.189 | 0.298 | 0.256 | 0.528 | 0.396 | 0.427 |
| *as* | 0,576 | 0,622 | 0,652 | 0,797 | 0,359 | 0,680 | 0,750 | 0,522 | 0,620 | 0,545 | 0,746 | 0,689 | 0,438 |
| *esp* | 0,315 | 0,456 | 0,389 | 0,624 | 0,269 | 0,645 | 0,709 | 0,536 | 0,705 | 0,593 | 0,673 | 0,600 | 0,669 |
| *cob* | 0,065 | 0,232 | 0,219 | 0,568 | 0,464 | 0,883 | 0,627 | 0,252 | 0,420 | 0,300 | 0,544 | 0,398 | 0,686 |
| *cpd* | 0,267 | 0,428 | 0,450 | 0,741 | 0,510 | 0,886 | 0,800 | 0,456 | 0,589 | 0,500 | 0,728 | 0,605 | 0,589 |
| *ccf* | 0,340 | 0,494 | 0,493 | 0,792 | 0,535 | 0,876 | 0,842 | 0,512 | 0,621 | 0,520 | 0,770 | 0,649 | 0,546 |

**Table S4**–Part D

| Variable | *van* A | *van* B | *van* C1 | *van* C2/C3 | *cyl*A | *hyl* | *ace* | *efa*A | *gel*E | *as* | *esp* | *cob* | *cpd* |
|---|---|---|---|---|---|---|---|---|---|---|---|---|---|
| *van*B | 0,459 | | | | | | | | | | | | |
| *van*C1 | -0,047 | 0,643 | | | | | | | | | | | |
| *van*C2/VC3 | -0,153 | -0,270 | -0,052 | | | | | | | | | | |
| *cyl*A | 0,243 | 0,363 | 0,446 | -0,008 | | | | | | | | | |
| *hyl* | 0,096 | 0,122 | 0,470 | 0,068 | 0,537 | | | | | | | | |
| *ace* | -0,023 | 0,510 | 0,807 | -0,082 | 0,553 | 0,709 | | | | | | | |
| *efa*A | 0,025 | 0,421 | 0,583 | -0,016 | 0,786 | 0,672 | 0,829 | | | | | | |
| *gel*E | 0,178 | 0,519 | 0,659 | -0,099 | 0,721 | 0,692 | 0,877 | 0,851 | | | | | |
| *as* | 0,065 | 0,456 | 0,666 | -0,022 | 0,822 | 0,557 | 0,653 | 0,811 | 0,764 | | | | |
| *esp* | 0,047 | 0,540 | 0,732 | -0,037 | 0,624 | 0,501 | 0,679 | 0,696 | 0,625 | 0,739 | | | |
| *cob* | 0,063 | 0,623 | 0,809 | -0,230 | 0,494 | 0,585 | 0,884 | 0,795 | 0,800 | 0,762 | 0,686 | | |

| | | | | | | | | | | | | | |
|---|---|---|---|---|---|---|---|---|---|---|---|---|---|
| *cpd* | -0,026 | 0,533 | 0,805 | -0,023 | 0,664 | 0,658 | 0,885 | 0,912 | 0,827 | 0,866 | 0,748 | 0,932 | |
| *ccf* | 0,042 | 0,556 | 0,741 | 0,001 | 0,734 | 0,641 | 0,856 | 0,950 | 0,834 | 0,877 | 0,747 | 0,905 | 0,984 |

**Table S5**. Number and percentage of antibiotic resistant enterococci in wastewater and river water

| Antibiotic | Sampling sites | Number (%) of resistant *Enterococci:* | | | | | | | |
|---|---|---|---|---|---|---|---|---|---|
| | | *faecium* | *faecalis* | *durans* | *avium* | *hirae* | *gallinarum* | *casseliflavus/ flavescens* | Other *Enterococcus spp* |
| AMP | HWW | 26(72.2) | 7(26.9) | 1(100) | 0.0 | 1(50.0) | 0 | 0.0 | 9(90) |
| | UWW | 6(21.4) | 0(0.0) | 0.0 | 0.0 | 0.0 | 0.0 | 0.0 | 1(8.3) |
| | TWW | 10(26.3) | 1(3.4) | 1(33.3) | 0.0 | 1(25.0) | 0.0 | 0.0 | 2(9.5) |
| | URW | 0.0 | 0.0 | 0.0 | 0.0 | 0.0 | 0.0 | 0.0 | 0.0 |
| | DRW | 1(25) | 0.0 | 0.0 | 0.0 | 0.0 | 1(20.0) | 0.0 | 1(16.6) |
| IPM | HWW | 26(72.2) | 8(30.8) | 1(100) | 1(14.3) | 1(50) | 0.0 | 0.0 | 8(80.0) |
| | UWW | 7(25.0) | 3(15.0) | 0.0 | 0.0 | 0.0 | 0.0 | 0.0 | 3(25.0) |
| | TWW | 10(26.3) | 8(27.6) | 1(33.3) | 0.0 | 1(25.0) | 0.0 | 0.0 | 2(9.5) |
| | URW | 0.0 | 0.0 | 0.0 | 0.0 | 0.0 | 0.0 | 0.0 | 0.0 |
| | DRW | 2(50) | 1(100) | 0.0 | 0.0 | 0.0 | 1 (20) | 0.0 | 3(50) |
| GEN | HWW | 22(61.1) | 14(53.8) | 1(100) | 2(28.6) | 0.0 | 0.0 | 1(100) | 8(80) |
| | UWW | 5(17.8) | 3(15.0) | 0.0 | 1(25.0) | 0.0 | 0.0 | 0.0 | 1(8.3) |
| | TWW | 9(23.7) | 11(37.9) | 1(33.3) | 1(50) | 1(25.0) | 0.0 | 0.0 | 1(4.8) |
| | URW | 0.0 | 0.0 | 0.0 | 0.0 | 0.0 | 0.0 | 0.0 | 0.0 |
| | DRW | 3(75) | 0.0 | 0.0 | 0.0 | 0.0 | 0.0 | 0.0 | 1(16.6) |
| S | HWW | 36(100) | 25(96.2) | 1(100) | 7(100) | 2(100) | 1(100) | 1(100) | 10(100) |
| | UWW | 22(78.6) | 17(85.0) | 0.0 | 3(75.0) | 1(100) | 0.0 | 0.0 | 7(58.3) |
| | TWW | 33(86.8) | 27(93.1) | 3(100) | 2(100) | 4(100) | 1(100) | 0.0 | 10(47.6) |
| | URW | 0.0 | 4(80) | 0.0 | 0.0 | 0.0 | 0.0 | 0.0 | 4(33.3) |
| | DRW | 3 (75) | 0.0 | 0.0 | 0.0 | 0.0 | 0.0 | 0.0 | 2(33.3) |
| TEC | HWW | 2(5.6) | 2(7.7) | 0.0 | 1(14.3) | 1(50) | 0.0 | 1(100) | 0.0 |
| | UWW | 6(21.4) | 4(20.0) | 0.0 | 1(25.0) | 0.0 | 1(33.3) | 0.0 | 9(75.0) |
| | TWW | 4(10.5) | 9(31) | 0.0 | 0.0 | 0.0 | 0.0 | 0.0 | 11(52.4) |
| | URW | 0.0 | 0.0 | 0.0 | 0.0 | 0.0 | 0.0 | 0.0 | 2(16.6) |
| | DRW | 4 (100) | 1(100) | 0.0 | 0.0 | 0.0 | 3(60) | 0.0 | 5(83.3) |
| VAN | HWW | 8(22.2) | 9(34.6) | 0.0 | 1(14.3) | 0.0 | 1(100) | 1(100) | 1(10) |
| | UWW | 15(53.6) | 14(70.0) | 0.0 | 0.0 | 0.0 | 3(100) | 0.0 | 6(50.0) |
| | TWW | 22(57.9) | 18(62.1) | 0.0 | 0.0 | 3(75.0) | 1(100) | 0.0 | 13(61.9) |
| | URW | 0.0 | 1 (20) | 0.0 | 0.0 | 0.0 | 0.0 | 0.0 | 2(16.6) |
| | DRW | 3 (75) | 1(100) | 0.0 | 0.0 | 0.0 | 3(60) | 0.0 | 5(83.3) |
| QD | HWW | 23(63.9) | 24(92.3) | 1(100) | 6(85.7) | 2(100) | 1(100) | 1(100) | 6(60) |
| | UWW | 20(71.4) | 17(85.0) | 0.0 | 4(100) | 1(100) | 1(33.3) | 0.0 | 11(91.7) |
| | TWW | 27(71.1) | 27(93.1) | 2(66.7) | 2(100) | 4(100) | 1(100) | 0.0 | 13(61.9) |
| | URW | 0.0 | 3(60) | 0.0 | 0.0 | 0.0 | 0.0 | 0.0 | 5(41.6) |
| | DRW | 3 (75) | 1(100) | 0.0 | 0.0 | 0.0 | 2(40) | 0.0 | 4(66.6) |
| TGC | HWW | 16(44.4) | 19(73.1) | 1(100) | 3(42.9) | 1(50) | 0.0 | 0.0 | 5(50) |
| | UWW | 12(42.9) | 7(35.0) | 0.0 | 2(50.0) | 1(100) | 0.0 | 0.0 | 4(33.3) |
| | TWW | 13(34.2) | 12(41.4) | 0.0 | 1(50) | 1(25.0) | 0.0 | 0.0 | 3(14.3) |
| | URW | 0.0 | 0.0 | 0.0 | 0.0 | 0.0 | 0.0 | 0.0 | 0.0 |
| | DRW | 0.0 | 0.0 | 0.0 | 0.0 | 0.0 | 1(20) | 0.0 | 5(83.3) |
| LZD | HWW | 23(63.9) | 18(69.2) | 1(100) | 3(42.9) | 2(100) | 1(100) | 1(100) | 5(50) |
| | UWW | 16(57.1) | 9(45.0) | 0.0 | 2(50.0) | 1(100) | 0.0 | 0.0 | 4(33.3) |
| | TWW | 25(65.8) | 19(65.5) | 2(66.7) | 2(100) | 2(50) | 1(100) | 0.0 | 6(28.6) |
| | URW | 0.0 | 1(20) | 0.0 | 0.0 | 0.0 | 0.0 | 0.0 | 4(33.3) |
| | DRW | 4(100) | 0.0 | 0.0 | 0.0 | 0.0 | 1(20) | 0.0 | 4(66.6) |
| NIT | HWW | 26(72.2) | 11(42.3) | 1(100) | 3(42.9) | 2(100) | 1(100) | 0.0 | 5(50) |
| | UWW | 13(46.4) | 9(45.0) | 0.0 | 2(50.0) | 1(100) | 0.0 | 0.0 | 6(50.0) |

| | | | | | | | | | |
|---|---|---|---|---|---|---|---|---|---|
| | TWW | 24(63.2) | 14(48.3) | 3(100) | 1(50) | 3(75.0) | 0.0 | 0.0 | 7(33.3) |
| | URW | 0.0 | 29(40) | 0.0 | 0.0 | 0.0 | 0.0 | 0.0 | 0.0 |
| | DRW | 3(75) | 1(100) | 0.0 | 0.0 | 0.0 | 3(60) | 0.0 | 2(33.3) |
| W5 | HWW | 32(88.9) | 23(88.5) | 1(100) | 4(57.1) | 2(100) | 1(100) | 1(100) | 9(90) |
| | UWW | 25(89.3) | 13(65.0) | 0.0 | 4(100)) | 1(100) | 0.0 | 0.0 | 12(100) |
| | TWW | 34(89.5) | 23(79.3) | 1(100) | 1(50) | 3(75.0) | 1(100) | 0.0 | 16(76.2) |
| | URW | 0.0 | 3(60) | 0.0 | 0.0 | 0.0 | 0.0 | 0.0 | 7(33.3) |
| | DRW | 4 (100) | 1(100) | 0.0 | 0.0 | 0.0 | 3(60) | 0.0 | 6(100) |
| CIP | HWW | 30(83.3) | 21(80.8) | 1(100) | 2(28.6) | 2(100) | 0.0 | 1(100) | 9(90) |
| | UWW | 17(60.7) | 8(40.0) | 0.0 | 2(50.0) | 1(100) | 2(66.7) | 0.0 | 7(58.3) |
| | TWW | 28(73.7) | 19(65.5) | 1(33.3) | 1(50) | 1(25.0) | 0.0 | 0.0 | 15(71.4) |
| | URW | 0.0 | 1(20) | 0.0 | 0.0 | 0.0 | 0.0 | 0.0 | 4(33.3) |
| | DRW | 1 (25) | 0.0 | 0.0 | 0.0 | 0.0 | 0.0 | 0.0 | 1(16.6) |
| DO | HWW | 0.0 | 3(11.5) | 0.0 | 0.0 | 0.0 | 0.0 | 0.0 | 0.0 |
| | UWW | 2(7.1) | 1(5.0) | 0.0 | 0.0 | 0.0 | 0.0 | 0.0 | 0.0 |
| | TWW | 14(36.8) | 8(27.6) | 0.0 | 0.0 | 0.0 | 0.0 | 0.0 | 0.0 |
| | URW | 0.0 | 0.0 | 0.0 | 0.0 | 0.0 | 0.0 | 0.0 | 2(16.6) |
| | DRW | 1(25) | 0.0 | 0.0 | 0.0 | 0.0 | 2(40.0) | 0.0 | 0.0 |

HWW – hospital waste water; UWW and TWW – untreated and treated wastewater; URW and DRW – upstream and downstream river water; AMP–ampicillin; IPM–imipenem; GEN–gentamicin; S–streptomycin; TEC–teicoplanin; VAN–vancomycin; QD–quinupristin/dalfopristin; TGC–tigecycline; LZD–linezolid; NIT–nitrofurantoin; W5–trimethoprim; CIP–ciprofloxacin; DO–doxycycline

**Table S6** Number and percentage of virulent enterococci in wastewater and river water

| Virulence factors | Sampling sites | Number (%) of virulent *Enterococci:* | | | | | | | |
|---|---|---|---|---|---|---|---|---|---|
| | | *faecium* | *faecalis* | *durans* | *avium* | *hirae* | *gallinarum* | *casseliflavus/ flavescens* | Other *Enterococcus spp* |
| *cyl* A | HWW | 9(25.0) | 11(42.3) | 0.0 | 2(28.6) | 0.0 | 1(100) | 1(100) | 1(10.0) |
| | UWW | 6(21.4) | 8(40.0) | 0.0 | 0.0 | 0.0 | 1(33.3) | 0.0 | 1(8.3) |
| | TWW | 9(23.7) | 12(41.4) | 0.0 | 1(50.0) | 0.0 | 1(10.0) | 0.0 | 3(14.3) |
| | URW | 0.0 | 2(40.0) | 0.0 | 0.0 | 0.0 | 0.0 | 0.0 | 1(8.3) |
| | DRW | 1(25.0) | 0.0 | 0.0 | 0.0 | 0.0 | 3(60.0) | 0.0 | 4(66.6) |
| *hyl* | HWW | 11(30.6) | 7(26.9) | 0.0 | 1(14.3) | 0.0 | 0.0 | 0.0 | 0.0 |
| | UWW | 7(25.0) | 5(25. 0) | 0.0 | 0.0 | 0.0 | 0.0 | 0.0 | 2(16.7) |
| | TWW | 13(34.2) | 15(51.7) | 1(33.3) | 0.0 | 0.0 | 0.0 | 0.0 | 6(28.6) |
| | URW | 0.0 | 2(40.0) | 0.0 | 0.0 | 0.0 | 0.0 | 0.0 | 3(25) |
| | DRW | 2(50.0) | 0.0 | 0.0 | 0.0 | 0.0 | 5(10.0) | 0.0 | 3(50.0) |
| *ace* | HWW | 0.0 | 8(30. 8) | 0.0 | 1(14.3) | 0.0 | 0.0 | 0.0 | 1(10.0) |
| | UWW | 8(28.6) | 15(75.0) | 0.0 | 0.0 | 0.0 | 2(66.7) | 0.0 | 0.0 |
| | TWW | 15(39.5) | 15(51.7) | 1(33.3) | 1(50.0) | 1(25. 0) | 0.0 | 0.0 | 1(4.8) |
| | URW | 0.0 | 2(40.0) | 0.0 | 0.0 | 0.0 | 0.0 | 0.0 | 1(8.3) |
| | DRW | 2(50.0) | 0.0 | 0.0 | 0.0 | 0.0 | 2(40.0) | 0.0 | 2(33.3) |
| *efa* A | HWW | 9(25.0) | 23(88.5) | 0.0 | 4(57.1) | 0.0 | 0.0 | 0.0 | 1(10.0) |
| | UWW | 13(46.4) | 15(75.0) | 0.0 | 3(75.0) | 0.0 | 2(66.7) | 0.0 | 2(16.7) |
| | TWW | 21(55.3) | 25(86.2) | 2(66.7) | 2(10.0) | 3(75.0) | 0.0 | 0.0 | 7(33.3) |
| | URW | 0.0 | 0.0 | 0.0 | 0.0 | 0.0 | 0.0 | 0.0 | 2(16.6) |
| | DRW | 3(75) | 1(10.0) | 0.0 | 0.0 | 0.0 | 3(60.0) | 0.0 | 2(33.3) |
| *gel* E | HWW | 3(8.3) | 13(50.0) | 0.0 | 1(14.3) | 0.0 | 0.0 | 0.0 | 1(10.0) |
| | UWW | 8(28.6) | 9(45.0) | 0.0 | 0.0 | 0.0 | 0.0 | 0.0 | 0.0 |
| | TWW | 14(36.8) | 17(58.6) | 2(66.7) | 1(50.0) | 0.0 | 0.0 | 0.0 | 1(4.8) |
| | URW | 0.0 | 2(40.0) | 0.0 | 0.0 | 0.0 | 0.0 | 0.0 | 1(8.3) |

| | | | | | | | | | |
|---|---|---|---|---|---|---|---|---|---|
| | DRW | 2(50.0) | 1(10.0) | 0.0 | 0.0 | 0.0 | 2(40.0) | 0.0 | 1(16.6) |
| *as* | HWW | 4(11.1) | 10.0(38.5) | 0.0 | 4(57.1) | 1(10.0) | 1(10.0) | 1(10.0) | 2(20.0) |
| | UWW | 4(14.3) | 6(30.0) | 0.0 | 0.0 | 1(10.0) | 0.0 | 0.0 | 0.0 |
| | TWW | 8(21.1) | 13(44.8) | 2(66.7) | 2(10.0) | 0.0 | 0.0 | 0.0 | 0.0 |
| | URW | 0.0 | 1(20.0) | 0.0 | 0.0 | 0.0 | 0.0 | 0.0 | 1(8.3) |
| | DRW | 2(50.0) | 0.0 | 0.0 | 0.0 | 0.0 | 0.0 | 0.0 | 0.0 |
| *esp* | HWW | 2(5.6) | 9(34.6) | 0.0 | 1(14.3) | 0.0 | 1(10.0) | 0.0 | 0.0 |
| | UWW | 6(21.4) | 1(5.0) | 0.0 | 1(25.0) | 0.0 | 0.0 | 0.0 | 1(8.3) |
| | TWW | 7(18.4) | 12(41.4) | 13(3.3) | 1(50.0) | 0.0 | 0.0 | 0.0 | 1(4.8) |
| | URW | 0.0 | 2(40.0) | 0.0 | 0.0 | 0.0 | 0.0 | 0.0 | 1(8.3) |
| | DRW | 2(50.0) | 1(10.0) | 0.0 | 0.0 | 0.0 | 4(80.0) | 0.0 | 0.0 |
| *cob* | HWW | 0.0 | 10.0(38.5) | 0.0 | 5(71.4) | 0.0 | 0.0 | 0.0 | 3(30.0) |
| | UWW | 10.0(35.7) | 9(45.0) | 0.0 | 1(25.0) | 0.0 | 0.0 | 0.0 | 1(8.3) |
| | TWW | 10.0(26.0)3) | 24(82.8) | 2(66.7) | 1(50.0) | 0.0 | 0.0 | 0.0 | 2(9.5) |
| | URW | 0.0 | 2(40.0) | 0.0 | 0.0 | 0.0 | 0.0 | 0.0 | 1(8.3) |
| | DRW | 3(75) | 0.0 | 0.0 | 0.0 | 0.0 | 1(20.0) | 0.0 | 1(16.6) |
| *cpd* | HWW | 4(11.1) | 20.0(76.9)) | 0.0 | 6(85.7) | 0.0 | 0.0 | 0.0 | 3(30.0) |
| | UWW | 13(46.6) | 14(70.0) | 0.0 | 1(25.0) | 0.0 | 1(33.3) | 0.0 | 2(16.7) |
| | TWW | 14(36.8) | 29(10.0) | 2(66.7) | 1(50.0) | 1(25.0) | 0.0 | 0.0 | 4(19.0) |
| | URW | 0.0 | 3(60.0) | 0.0 | 0.0 | 0.0 | 0.0 | 0.0 | 1(8.3) |
| | DRW | 2(50.0) | 0.0 | 0.0 | 0.0 | 0.0 | 4(80.0) | 0.0 | 1(16.6) |
| *ccf* | HWW | 7(19.4) | 22(84.6) | 0.0 | 7(10.0) | 0.0 | 1(10.0) | 0.0 | 3(30.0) |
| | UWW | 16(57.1) | 14(70.0) | 0.0 | 2(50.0) | 0.0 | 2(66.7) | 0.0 | 2(16.7) |
| | TWW | 15(39.5) | 29(10.0) | 2(66.7) | 1(50.0) | 3(75.0) | 1(10.0) | 0.0 | 3(14.3) |
| | URW | 0.0 | 3(60.0) | 0.0 | 0.0 | 0.0 | 0.0 | 0.0 | 2(16.6) |
| | DRW | 3(75) | 0.0 | 0.0 | 0.0 | 0.0 | 4.80.0) | 0.0 | 1(16.6) |

virulence factors: cytolysin (cylA), aggregation substance (AS), gelatinase (gel E), hyaluronidase (hyl), endocarditis antigen (efaA), factor encoding surface protein (esp), and sex pheromones (cob, cpd and ccf); HWW – hospital waste water; UWW and TWW – untreated and treated wastewater; URW and DRW – upstream and downstream river water;

## References


1. Ke, D.; Picard, F.J.; Martineau, F.; Menard, C.; Roy, P.H.; Ouellette, M. Bergeron M.G. Development of a PCR assay for rapid detection of enterococci. *J. Clin. Microbiol.* **1999**, 37, 3497 *– 3503.* https://doi.org/10.1128/JCM.37.11.3497-3503.1999.
2. Deasy, B.M.; Rea, M.C.; Fitzgerald, G.F.; Cogan, T.M.; Beresford, T.P. A rapid PCR based method to distinguish between *Lactococcus* and *Enterococcus*. *Syst. Appl Microbiol*. **2000**, 23, 510 – 522. https://doi.org/10.1016/S0723-2020(00)80025-9.
3. Kariyama, R.; Mitsuhata, R.,; Chow, J.W.; Clewell, D.B.; Kumon, H. Simple and reliable multiplex PCR assay for surveillance isolates of vancomycin resistant enterococci. *J. Clin. Microbiol.* **2000**, 38, 3092 – 3095. https://doi.org/10.1128/JCM.38.8.3092-3095.2000.
4. Cheng, S.; McCleskey, F.K; Gress, M.J.; Petroziello, J.M.; Liu, R.; Namdari, H.; Beninga, K.; Salmen, A.; DelVecchio, V.G. A PCR assay for identification of *Enterococcus faecium*. *J. Clin. Microbiol.* **1997**, 35, 1248 – 1250. https://doi.org/10.1128/JCM.35.5.1248-1250.1997.
5. Jackson, C.R.; Fedorka-Cray, P.J.; Barrett, J.B. Use of a genus- and species-specific multiplex PCR for identification of enterococci. *J. Clin. Microbiol.* **2004**, 42, 3558 – 3565. https://doi.org/10.1128/JCM.42.8.3558–3565.2004.

6. Dutka-Malen, S.; Evers, S.; Courvalin, P. Detection of glycopeptide resistance genotypes and identification to the species level of clinically relevant enterococci by PCR. *J. Clin. Microbiol.* **1995**, 33, 24 – 27. https://doi.org/10.1128/JCM.33.1.24-27.1995.
7. Van de Klundert, J.A.M.; Vliegenthart, J.S. PCR detection of genes coding for aminoglycoside-modifying enzymes. In: Persing D.H., Smith T.F., Tenover F.C., White T.J., editors. Diagnostic molecular microbiology. Washington, D.C.: ASM, **1993**, 547 – 552.
8. Vankerckhoven, V.; Van Autgaerden, T.; Vael, C.; Lammens, C.; Chapelle, S.; Rossi, R.; Jabes, D.; Goossens, H. Development of a Multiplex PCR for the Detection of asa1, gelE, cylA, esp, and hyl Genes in Enterococci and Survey for Virulence Determinants among European Hospital Isolates of *Enterococcus faecium*. *J. Clin. Microbiol.* **2004**, 42(10), 4473 – 4479. https://doi.org/10.1128/JCM.42.10.4473-4479.2004.
9. Mannu, L.; Paba, A.; Daga, E.; Comunian, R.; Zanetti, S.; Duprè, I.; Sechi, L.A. Comparison of the incidence of virulence determinants and antibiotic resistance between *Enterococcus faecium* strains of dairy, animal and clinical origin. *Int. J. Food Microbiol*. **2003**, 88(2-3), 291 – 304. https://doi.org/10.1016/S0168-1605(03)00191-0.
10. Shankar, V.; Baghdayan, A.S.; Huycke, M.M.; Lindahl, G.; Gilmore, M.S. Infection-derived *Enterococcus faecalis* strains are enriched in *esp*, a gene encoding a novel surface protein. *Infect. Immun.*. **1999**, 67, 193 – 200. https://doi.org/10.1128/IAI.67.1.193-200.1999.
11. Eaton, T.J.; Gasson, M.J. Molecular screening of *Enterococcus* virulence determinants and potential for genetic exchange between food and medical isolates. *Appl. Environ. Microbiol.* **2001**, 67, 1628 – 1635. https://dx.doi.org/10.1128%2FAEM.67.4.1628-1635.2001.
